# Associations of Filaggrin Gene Loss-of-Function Variants and Human Papillomavirus-Related Cancer and Pre-Cancer in Danish Adults

**DOI:** 10.1371/journal.pone.0099437

**Published:** 2014-06-06

**Authors:** Tea Skaaby, Lise Lotte N. Husemoen, Torben Jørgensen, Jeanne D. Johansen, Torkil Menné, Pal B. Szecsi, Steen Stender, Peter Bager, Jacob P. Thyssen, Allan Linneberg

**Affiliations:** 1 Research Centre for Prevention and Health, Glostrup University Hospital, Glostrup, Denmark; 2 Faculty of Health Science, University of Copenhagen, Copenhagen, Denmark; 3 Faculty of Medicine, Alborg University, Alborg, Denmark; 4 Department of Clinical Biochemistry, Copenhagen University Hospital Gentofte, Hellerup, Denmark; 5 Department of Epidemiology Research, National Center for Health Data and Disease Control, Copenhagen, Denmark; 6 National Allergy Research Centre, Department of Dermato-Allergology, Copenhagen University Hospital Gentofte, Hellerup, Denmark; 7 Department of Clinical Experimental Research, Glostrup University Hospital, Glostrup, Denmark; 8 Department of Clinical Medicine, Faculty of Health and Medical Sciences, University of Copenhagen, Copenhagen, Denmark; State University of Maringá/Universidade Estadual de Maringá, Brazil

## Abstract

**Purpose:**

Filaggrin proteins are expressed in the skin, oral cavity, oesophagus, and cervical mucose. Loss-of-function mutations in the filaggrin gene (*FLG*) reduce filaggrin expression and cause an impaired skin barrier function. We hypothesized that *FLG* mutation carriers would be more susceptible to human papillomavirus (HPV) infection and thus a higher risk of HPV-related cancer and pre-cancer. We investigated the association of the *FLG* genotype with incidence of HPV-related cancer of cervix, vagina, vulva, penis, anus and head and neck, and pre-cancer of the cervix.

**Methods:**

We included 13,376 persons from four population-based studies conducted in the same background population in Copenhagen, Denmark. Participants were genotyped for the most common *FLG* mutations in Europeans. Information on cancer was obtained from The Danish Cancer Registry until 11 July 2011.

**Results:**

There were 489 cases of prevalent and 97 cases of incident HPV-related cancer and pre-cancer (median follow-up 11.5 years). There was a statistically significant association between *FLG* genotype and incident HPV-related cancer and pre-cancer with a hazard ratio, HR = 2.1 (95% confidence intervals, CI: 1.2, 3.7) for *FLG* mutation carriers vs. wild types.

**Conclusions:**

*FLG* loss-of-function mutations were associated with higher incidence of HPV-related cancers and pre-cancers that are potentially screening and vaccine preventable.

## Introduction

Human papillomavirus (HPV) is a DNA virus infecting keratinocytes or cells in mucous membranes. Most known HPV types are largely harmless, some cause warts, whereas oncogenic types can cause pre-cancer and cancer of the cervix, vulva, vagina, penis, anus and a subgroup of head and neck cancers [1]. Precancerous lesions such as dysplasia and carcinoma *in situ* (CIS) of the cervix are common among women and if left untreated may lead to cancer. Cervical cancer is the fourth highest contributor to women cancer mortality worldwide and the second most common cause of cancer mortality among women in Africa [2]. Anal cancer primarily affects gay and bisexual men and is rare but increasing [1], [3].

With the introduction of organized cervical cytological screening programs, cervical cancer incidence has been substantially reduced [4]. The developments in prophylactic HPV vaccination have renewed the interest in HPV-related cancers and cell changes. Vaccination against the two most important oncogenic HPV types (type 16 and 18) lowers the risk of anal, vulva, vaginal and penile infections with the two HPV types and decreases the risk of precancerous cervical lesions [5], [6]. These two HPV types are likely responsible for 70% of cervical cancers and most of the non-cervical HPV-related cancers [1].

The epidermal layer of the skin provides a barrier against environmental exposures including microorganisms. Filaggrin proteins display structural and physiological functions in the skin but is also expressed in the oral cavity, cervix, endometrium, and vagina [7], [8]. While the role of filaggrin outside the skin is largely unknown, the degradation products keep the epidermis acidic thereby preventing the colonization of microorganisms. Loss-of-function mutations in the filaggrin gene (*FLG*) reduce epidermal filaggrin levels and are among the most frequent known single-gene defects [9], [10]. *FLG* loss-of-function mutations are strong genetic risk factors for atopic dermatitis in particular, but also carry a higher risk of rhinitis, asthma, and food allergies in the context of atopic dermatitis [7].

*FLG* loss-of-function mutations may lead to a greater susceptibility to HPV-related cancer and pre-cancer due to an impaired barrier function and atopic dermatitis [7], [11]; elevated pH of the stratum corneum [7]; and a low grade skin inflammation [12]–[14]. We investigated the association of the *FLG* genotype and HPV-related cancer of cervix, vagina, vulva, penis, anus and head and neck and pre-cancer of the cervix according to the *International Classification of Diseases* (ICD) in four population-based studies.

## Material and Methods

### Ethics statement

Participants gave their informed written consent, and the studies were approved by the Ethics Committee of Copenhagen and the Danish Data Protection Agency. The recommendations of the Declaration of Helsinki were followed.

### Study populations

We included the four population based studies, Monica10, Inter99, Health2006, and Allergy98, where the former three are recruited from the Danish Central Personal Register as random samples of the population in the southern part of the former Copenhagen County. The studies included questionnaires, physical examinations, and blood tests.

The Monica10 study was conducted in 1993-94 and included 2,656 persons of Danish origin (4,130 invited) between 40–71 years and had a participation rate of 64.3% [15].

The Inter99 study conducted in 1999–2001 included 6,784 persons aged 30–60 years [16]. The Inter99 study was a population-based randomized controlled trial (CT00289237, ClinicalTrials.gov) investigating the effects of lifestyle intervention on cardiovascular disease. The baseline participation rate was 52.5%. Details on the study and the intervention program have been described elsewhere [16]. Only participants with a Northern European origin were included in the current study. Both current and potential former nationalities of participants and their parents were considered (information from registries and self-reported questionnaires). A Northern European origin was defined as a Danish, Norwegian, Swedish, Icelandic, or Faroese nationality.

In the Health2006 study, a sample of 7,931 Danish citizens aged 18 to 69 years, born in Denmark, was invited to a general health examination [17]. A total of 3,471 (43.8%) individuals were examined between June 2006 and June 2008.

The Copenhagen Allergy study began in 1990 and included a group of persons randomly selected from the general population and a selected group of persons with allergic respiratory symptoms (recruited from a random sample of the general population by a screening questionnaire). We used data from the follow-up study in 1997–1998 (Allergy98) where a total of 1,966 persons aged 15–77 years with Danish nationality were invited for a health examination. A total of 1,216 (61.9%) participated [18].

**Figure 1:**
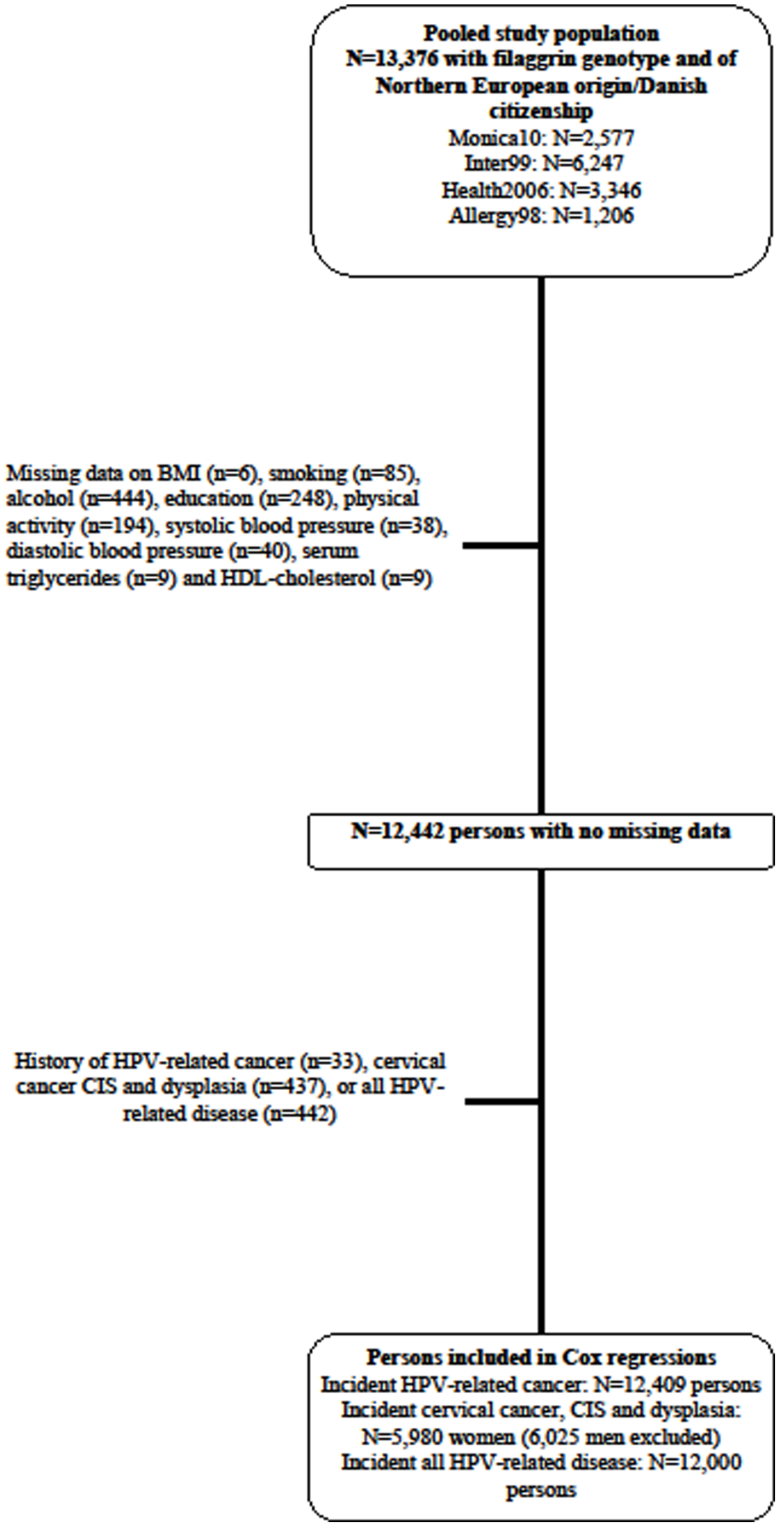
Flowchart

We included a total of 13,376 persons with *FLG* genotype: 2,577, 6,247, 1,206, and 3,346 participants from the Monica10, the Inter99, the Allergy98, and the Health2006 study, respectively (Figure 1).

### FLG genotyping

Individual regions covering the two most common null mutations of the *FLG*, R501X and 2282del4 (in the Inter99 and Health2006 studies also a region covering the R2447X mutation) were amplified from genomic DNA by allele-specific and asymmetric PCR using DNA tagged primers in all four studies. The obtained PCR products were hybridized to MagPlex C microbeads (Luminex, Austin, Texas) carrying the same tags as DNA probes [19]. Microbeads were subsequently analyzed on a Bio-Plex 200 (Bio-Rad Laboratories, Hercules, Calif). The filaggrin mutation analysis is ISO 15189 accredited. Samples also available as DNA were genotyped for *FLG* mutations. The genotyping success rates were: Monica10: 99.96%, Allergy98: >99%, Inter99: 99.95%, Health2006: 99.40%.

The gene frequencies were: Monica10: (R501X: 2.8%, 2282del4: 4.8%), Allergy98: (R501X: 3.4%, 2282del4: 3.8%), Inter99: (R501X: 3.4%, 2282del4: 4.4%, R2447X: 1.0%), and Health2006: (R501X: 3.4%, 2282del4: 4.7%, R2447X: 0.9%). All three *FLG* genotypes were in Hardy-Weinberg equilibrium (tested by the Hardy-Weinburg equilibrium test) [20]. *FLG* genotype was categorized as: no *FLG* mutations; or at least one *FLG* mutation (heterozygotes, homozygotes and compound homozygotes).

### Registry-based diagnoses

People living in Denmark have since 1968 been assigned a unique and permanent personal civil registration number which enables linkage of data from complete national registers on an individual level. Information on cancer diagnoses was obtained from the Danish Cancer Register [21], [22] according to the *International Classification of Diseases* (ICD). Reporting to the Cancer Registry has been mandatory since 1987. From 1943 to 1978 the Registry was classified according to the modified ICD-7, and from 1978 and onwards the diagnoses were coded in accordance with the ICD-10 [21]. Information on death from any cause and emigration status was obtained from the Danish Civil Registration System [23]. Participants were followed until 11 July 2011.

We included the cancers most strongly associated with HPV [24]: Cancer, cervix (ICD-7: 1711, 1712, ICD-10: C53), CIS, cervix (ICD-7: 5710, 5711, ICD-10: D06), dysplasia, cervix (ICD-7: 5712, ICD-10: N87), cancer, head and neck, subset (ICD-7: 1410, 1411, 1418, 1450, 1442, 1458, 1480, ICD-10: C01.9, C02.4, C02.8, C09.0, C09.1, C09.8, C09.9, C10.2, C10.8, C10.9, C14.0, C14.2, C14.8), cancer, vulva and vagina (ICD-7: 1760, 1761, 1762, 1763, 1765, ICD-10: C51, C52), cancer, penis (ICD-7: 1790, ICD-10: C60), and cancer, anus (ICD-7: No sufficiently specific ICD-7 code, ICD-10: C21.0–C21.8).

We defined three end points: cervical cancer and pre-cancer (Cervical dysplasia, CIS and cancer); all HPV-related cancers (subsets of cancers of head and neck, cervix, vulva, vagina, penis and anus as defined above); and all HPV-related cancers and pre-cancers (subsets of cancers of head and neck, cervix, vulva, vagina, penis and anus and cervical dysplasia and CIS).

Prevalent cancer and pre-cancer were defined as a diagnosis of the cancer and pre-cancer of interest before baseline, i.e. the date of the health examination (both ICD-7 and ICD-10 codes). Incident cancer and pre-cancer were defined as a diagnosis of the cancer and pre-cancer of interest during follow-up (only ICD-10 codes) among those without a diagnosis of the cancer or pre-cancer of interest at baseline. All time cancer and pre-cancer were defined as either prevalent or incident (or both) cancer and pre-cancer.

### Other covariates

The questionnaires gave information on the covariates physical activity during leisure time (sedentary, light, or moderate/vigorous); education/vocational training (only basic education, education including students); alcohol consumption (drinks per week); smoking habits (daily smokers; or never, former and occasional smokers).

Height and weight were measured without shoes and light clothes, and body mass index (BMI) was calculated as weight divided by height squared (kg/m^2^). We used the average of two blood pressure measurements. In the Monica10, Inter99 and Health2006 study, serum triglycerides and HDL-cholesterol were measured from fasting blood samples using enzymatic colorimetric methods (Roche, Mannheim, Germany). In Allergy98, HDL-cholesterol and triglycerides were determined from non-fasting blood samples using the VITROS 950 automatic analyzer (Johnson & Johnson, Langhorne, Pa., USA).

### Statistical analyses

The analyses were performed with SAS, version 9.2 (SAS Institute Inc. Cary, NC USA). All p-values were two-sided, and p<0.05 were considered statistically significant. Table 1 shows the baseline characteristics expressed as % (number) or mean (standard deviation, SD) according to study population and *FLG* genotype. Table 2 displays covariates/baseline characteristics according to all time HPV-related disease. Table 3 shows the distribution of prevalent and incident cancers and pre-cancers in the 4 studies.

**Table 1:** Baseline characteristics according to study population and *FLG* genotype.

Study population	Monica10, % (n); *mean (SD)*		Allergy98, % (n); *mean (SD)*		Inter99, % (n); *mean (SD)*		Health2006, % (n); *mean (SD)*	
*FLG* genotype	Wild type	Mutation*	Wild type	Mutation*	Wild type	Mutation*	Wild type	Mutation*
**Persons**	92.5 (2,383)	7.5 (194)	92.9 (1,120)	7.1 (86)	91.2 (5,696)	8.8 (551)	91.1 (3,047)	8.9 (299)
**Age** (years)	*55.2 (10.8)*	*56.1 (10.0)*	*39.9 (15.1)*	*42.1 (15.2)*	*46.2 (7.9)*	*46.5 (8.0)*	*49.4 (13.0)*	*49.2 (13.2)*
*P-value*		*P = 0.174*		*P = 0.185*		*P = 0.539*		*P = 0.107*
**Male gender**	92.8 (1,193)	7.2 (93)	92.9 (512)	7.1 (39)	90.6 (2,754)	9.4 (285)	91.2 (1,365)	8.8 (132)
**Female gender**	92.2 (1190)	7.8 (101)	92.8 (608)	7.2 (47)	91.7 (2942)	8.3 (266)	91.0 (1682)	9.0 (167)
P-value		P = 0.569		P = 0.948		P = 0.130		P = 0.829
**Alcohol** (drinks/week)	*10.0 (12.3)*	*9.6 (12.4)*	*6.8 (8.0)*	*7.2 (8.9)*	*10.3 (12.6)*	*11.5 (19.2)*	*9.6 (10.1)*	*9.9 (10.9)*
*P-value*		*P = 0.214*		*P = 0.854*		*P = 0.228*		*P = 0.765*
**Education**	92.8 (1778)	7.2 (137)	92.3 (791)	7.7 (66)	91.2 (4669)	8.8 (450)	91.4 (2611)	8.6 (247)
**No education**	91.4 (604)	8.6 (57)	94.2 (325)	5.8 (20)	90.9 (848)	9.1 (85)	89.1 (392)	10.9 (48)
P-value		P = 0.217		P = 0.247		P = 0.782		P = 0.121
**BMI** (kg/m^2^)	*25.9 (4.2)*	*26.2 (4.1)*	*25.6 (4.6)*	*25.9 (4.1)*	*26.3 (4.6)*	*26.1 (4.5)*	*25.9 (4.7)*	*26.1 (5.1)*
*P-value*		*P = 0.209*		*p = 0.325*		*P = 0.140*		*P = 0.768*
**Sedentary PA**	93.1 (499)	6.9 (37)	92.3 (289)	7.7 (24)	91.8 (1,168)	8.2 (105)	89.9 (550)	10.1 (62)
**Light PA**	92.0 (1326)	8.0 (115)	92.1 (556)	7.9 (48)	90.8 (3479)	9.2 (352)	91.5 (1838)	8.5 (170)
**Moderate/vigorous PA**	93.0 (518)	7.0 (39)	95.4 (270)	4.6 (13)	91.9 (949)	8.1 (84)	91.3 (631)	8.7 (60)
P-value		P = 0.624		P = 0.173		P = 0.412		P = 0.441
**Never/former smokers**	91.7 (1296)	8.3 (117)	92.3 (741)	7.7 (62)	90.8 (3632)	9.2 (369)	91.4 (2343)	8.6 (220)
**Daily smokers**	93.3 (1,080)	6.7 (77)	94.0 (377)	6.0 (24)	91.9 (2,023)	8.1 (179)	89.8 (674)	10.2 (77)
P-value		P = 0.121		P = 0.270		P = 0.146		P = 0.159
**Systolic blood pressure**	*129.5 (19.3)*	*127.6 (18.2)*	*128.7 (17.8)*	*131.8 (18.5)*	*130.6 (17.6)*	*130.9 (17.0)*	*130.5 (17.9)*	*130.7 (17.0)*
*P-value*		*P = 0.215*		*P = 0.158*		*P = 0.541*		*P = 0.685*
**Diastolic blood pressure**	*82.2 (10.5)*	*81.0 (10.9)*	*79.3 (11.4)*	*82.1 (12.4)*	*82.6 (11.4)*	*82.3 (11.0)*	*81.7 (10.7)*	*82.9 (10.4)*
*P-value*		*P = 0.095*		*P = 0.070*		*P = 0.954*		*P = 0.059*
**Triglycerides**	*1.5 (1.0)*	*1.4 (1.0)*	*1.7 (1.2)*	*1.6 (0.9)*	*1.3 (1.3)*	*1.3 (1.9)*	*1.3 (1.0)*	*1.3 (0.7)*
*P-value*		*P = 0.689*		*P = 0.915*		*P = 0.100*		*P = 0.772*
**HDL-cholesterol**	*1.4 (0.4)*	*1.5 (0.5)*	*1.6 (0.4)*	*1.6 (0.4)*	*1.4 (0.4)*	*1.5 (0.4)*	*1.5 (0.4)*	*1.5 (0.4)*
*P-value*		*P = 0.469*		*P = 0.002*		*P = 0.014*		*P = 0.879*

*Mean (SD) and p-values from the Kruskal-Wallis test are in italic.*

^*^ One or more FLG loss-of-function mutations

Abbreviations: BMI, body mass index; SD, standard deviation; HDL-cholesterol, high-density lipoprotein cholesterol; *FLG*, filaggrin gene; PA, physical activity

**Table 2:** Covariates according to human papilloma virus related disease* at any time.

	% (n); *Mean (SD)*	% (n); *Mean (SD)*	P-value
Characteristics	No HPV-related diagnosis (n = 12,790)	HPV-related diagnosis (n = 586)	Chi-square test or *Kruskal Wallis test*
Study			0.065
Monica10	96.3 (2,481)	3.7 (96)	
Inter99	95.5 (5,965)	4.5 (282)	
Health2006	95.1 (3,181)	4.9 (165)	
Allergy98	96.4 (1,163)	3.6 (43)	
Gender			<0.0001
Male	99.7 (6,355)	0.3 (18)	
Female	91.9 (6,435)	8.1 (568)	
Age, years	*48.3 (11.5)*	*47.2 (10.6)*	*0.026*
Education			0.137
No	95.0 (2,261)	5.0 (118)	
Yes	95.7 (10,290)	4.3 (459)	
Body mass index, kg/m^2^	*26.1 (4.5)*	*25.2 (5.1)*	*<0.0001*
Physical activity			0.0003
Sedentary	94.6 (2,585)	5.4 (149)	
Light	95.6 (7,533)	4.4 (351)	
Moderate/vigorous	96.8 (2,483)	3.2 (81)	
Never/former smokers	96.5 (8,470)	3.5 (310)	
Daily smokers	93.9 (4,236)	6.1 (275)	<0.0001
Alcohol, drinks/week	*9.9 (12.1)*	*7.3 (8.8)*	*<0.0001*
Systolic blood pressure	*130.4 (17.9)*	*125.8 (18.3)*	*<0.0001*
Diastolic blood pressure	*82.1 (11.1)*	*79.3 (11.2)*	*<0.0001*
Triglycerides	*1.4 (1.2)*	*1.3 (0.9)*	*0.001*
HDL-cholesterol	*1.5 (0.4)*	*1.6 (0.4)*	*<0.0001*

*Mean (SD) and p-values from the Kruskal-Wallis test are in italic.*

^*^ Cervical cancer, carcinoma in situ and dysplasia and human papilloma virus related cancers of head and neck, vulva and vagina, penis and anus.

**Table 3:** Distribution of prevalent and incident human papilloma virus related disease according to study population and *FLG* genotype.

		HPV-related cancer*, % (n/n_total_)**		Cervical cancer, CIS and dysplasia***, % (n/n_total_)****	
*FLG* genotype		Wild type	Loss-of-function mutation	Wild type	Loss-of-function mutation
Total	Prevalent	0.3 (33/12246)	0.1 (1/1130)	6.8 (438/6422)	7.9 (46/581)
	Incident	0.2 (21/12246)	0.4 (4/1130)	1.0 (62/6422)	1.7 (10/581)
Monica10	Prevalent	0.3 (7/2383)	0.5 (1/194)	6.1 (72/1190)	7.9 (8/101)
	Incident	0.3 (7/2383)	1.0 (2/194)	0.6 (7/1190)	1.0 (1/101)
Allergy98	Prevalent	0.4 (4/1120)	0 (0/86)	3.8 (23/608)	4.3 (2/47)
	Incident	0.1 (1/1120)	0 (0/86)	2.0 (12/608)	4.3 (2/47)
Inter99	Prevalent	0.2 (13/5696)	0 (0/551)	7.4 (219/2942)	8.6 (23/266)
	Incident	0.1 (8/5696)	0.2 (1/551)	0.9 (26/2942)	1.5 (4/266)
Health2006	Prevalent	0.3 (9/3047)	0 (0/299)	7.4 (124/1682)	7.8 (13/167)
	Incident	0.2 (5/3047)	0.3 (1/299)	1.0 (17/1682)	1.8 (3/167)

^*^ HPV-related cancers include cancer, cervix (ICD-7: 1711, 1712, ICD-10: C53), cancer, head and neck, subset (ICD-7: 1410, 1411, 1418, 1450, 1442, 1458, 1480, ICD-10: C01.9, C02.4, C02.8, C09.0, C09.1, C09.8, C09.9, C10.2, C10.8, C10.9, C14.0, C14.2, C14.8), cancer, vulva and vagina (ICD-7: 1760, 1761, 1762, 1763, 1765, ICD-10: C51, C52), cancer, penis (ICD-7: 1790, ICD-10: C60), and cancer, anus (ICD-7: No sufficiently specific ICD-7 code, ICD-10: C21.0-C21.8).

^**^ Of the whole population, i.e. both men and women

^***^ Cervical cancer, CIS and dysplasia include cancer, cervix (ICD-7: 1711, 1712, ICD-10: C53), CIS, cervix (ICD-7: 5710, 5711, ICD-10: D06), and dysplasia, cervix (ICD-7: 5712, ICD-10: N87).

^****^ Of women

Abbreviations: CIS, carcinoma in situ; HPV, human papillomavirus; *FLG*, filaggrin gene

Data from the four cohorts were pooled. The associations between *FLG* genotype and prevalent and all-time cervical cancer and pre-cancer, HPV-related cancer and all HPV-related cancer and pre-cancer were analyzed with multivariable logistic regression analyses (table 4). The estimates are presented as odds ratios (OR) and 95% confidence intervals (CI). Multivariable Cox regression analyses were used to determine the association of *FLG* genotype and the incidence of HPV-related cancer, cervical cancer, CIS and dysplasia, and all HPV-related cancer and pre-cancer (table 5). We used age as underlying time axis and delayed entry which means that persons enter the analysis at their age at baseline and exit the analysis at their event or censoring age. The few participants lost to follow-up (emigrated or disappeared) contributed to the risk time until the date of their last registered activity. Persons with a diagnosis of cancer and pre-cancer at baseline were excluded in the analyses of all incident HPV-related cancer and pre-cancer. Estimates are presented as hazard ratios (95% CI).

**Table 4:** Odds ratios and 95% confidence intervals for the associations between *FLG* genotype and prevalent and all time HPV-related disease.

	Events (persons included)	Model 1$OR (95% CI)	Model 2& OR (95% CI)
**Prevalent disease**			
HPV-related cancer*	33 (12,442)		
*FLG* loss-of-function mutation vs. wild type		0.3 (0.05, 2.4)	0.3 (0.04, 2.4)
		P = 0.274	P = 0.269
Cervical cancer, CIS and dysplasia**	437 (6,417)		
*FLG* loss-of-function mutation vs. wild type		1.2 (0.9, 1.7)	1.2 (0.9, 1.7)
		P = 0.285	P = 0.256
All HPV-related disease***	442 (12,442)		
*FLG* loss-of-function mutation vs. wild type		1.2 (0.9, 1.6)	1.2 (0.9, 1.7)
		P = 0.324	P = 0.294
**All time disease (prevalent and incident)**			
HPV-related cancer*	55 (12,442)		
*FLG* loss-of-function mutation vs. wild type		1.1 (0.4, 2.7)	1.1 (0.4, 2.7)
		P = 0.875	P = 0.885
Cervical cancer, CIS and dysplasia**	506 (6,417)		
*FLG* loss-of-function mutation vs. wild type		1.3 (1.0, 1.8)	1.3 (0.98, 1.8)
		P = 0.081	P = 0.068
All HPV-related disease***	534 (12,442)		
*FLG* loss-of-function mutation vs. wild type		1.3 (1.0, 1.8)	1.4 (1.0, 1.8)
		P = 0.050	P = 0.040

$ Adjusted for study, gender and age. Not for cervical disease where only women were included.

& Further adjusted for education, physical activity, smoking habits, alcohol intake, body mass index, systolic and diastolic blood pressure, serum triglycerides and HDL-cholesterol.

^*^ HPV-related cancers include cancer, cervix (ICD-7: 1711, 1712, ICD-10: C53), cancer, head and neck, subset (ICD-7: 1410, 1411, 1418, 1450, 1442, 1458, 1480, ICD-10: C01.9, C02.4, C02.8, C09.0, C09.1, C09.8, C09.9, C10.2, C10.8, C10.9, C14.0, C14.2, C14.8), cancer, vulva and vagina (ICD-7: 1760, 1761, 1762, 1763, 1765, ICD-10: C51, C52), cancer, penis (ICD-7: 1790, ICD-10: C60), and cancer, anus (ICD-7: No sufficiently specific ICD-7 code, ICD-10: C21.0-C21.8).

^**^ Cervical cancer, CIS and dysplasia include cancer, cervix (ICD-7: 1711, 1712, ICD-10: C53), CIS, cervix (ICD-7: 5710, 5711, ICD-10: D06), and dysplasia, cervix (ICD-7: 5712, ICD-10: N87).

^***^ All HPV-related disease includes cancer, head and neck, subset (ICD-7: 1410, 1411, 1418, 1450, 1442, 1458, 1480, ICD-10: C01.9, C02.4, C02.8, C09.0, C09.1, C09.8, C09.9, C10.2, C10.8, C10.9, C14.0, C14.2, C14.8), cancer, vulva and vagina (ICD-7: 1760, 1761, 1762, 1763, 1765, ICD-10: C51, C52), cancer, penis (ICD-7: 1790, ICD-10: C60), cancer, anus (ICD-7: No sufficiently specific ICD-7 code, ICD-10: C21.0-C21.8), cancer, cervix (ICD-7: 1711, 1712, ICD-10: C53), CIS, cervix (ICD-7: 5710, 5711, ICD-10: D06), and dysplasia, cervix (ICD-7: 5712, ICD-10: N87).

Abbreviations: CI, confidence interval; OR, odds ratio, CIS, carcinoma in situ; HPV, human papillomavirus.

**Table 5:** Hazard ratios and 95% confidence intervals for the associations between *FLG* genotype and incident HPV-related disease.

	Events (persons included)	Model 1$ HR (95% CI)	Model 2& HR (95% CI)
HPV-related cancer*	22 (12,409)		
*FLG* wild type		1 (reference)	1 (reference)
*FLG* loss-of-function mutation		2.4 (0.8, 7.0)	2.4 (0.8, 7.1)
		P = 0.114	P = 0.122
Cervical cancer, CIS and dysplasia**	69 (5,980)		
*FLG* wild type		1 (reference)	1 (reference)
*FLG* loss-of-function mutation		2.0 (1.0, 3.8)	2.0 (1.0, 4.0)
		P = 0.050	P = 0.038
All HPV-related disease***	92 (12,000)		
*FLG* wild type		1 (reference)	1 (reference)
*FLG* loss-of-function mutation		2.0 (1.2, 3.6)	2.1 (1.2, 3.7)
		P = 0.014	P = 0.011

Complete case analysis. Persons suffering from the cancer or disease of interest at baseline were excluded.

$ Adjusted for study and gender (age is underlying time axis). Not for cervical disease where only women were included.

& Further adjusted for education, physical activity, smoking habits, alcohol intake, body mass index, systolic and diastolic blood pressure, serum triglycerides and HDL-cholesterol.

^*^ HPV-related cancers include cancer, cervix (ICD-7: 1711, 1712, ICD-10: C53), cancer, head and neck, subset (ICD-7: 1410, 1411, 1418, 1450, 1442, 1458, 1480, ICD-10: C01.9, C02.4, C02.8, C09.0, C09.1, C09.8, C09.9, C10.2, C10.8, C10.9, C14.0, C14.2, C14.8), cancer, vulva and vagina (ICD-7: 1760, 1761, 1762, 1763, 1765, ICD-10: C51, C52), cancer, penis (ICD-7: 1790, ICD-10: C60), and cancer, anus (ICD-7: No sufficiently specific ICD-7 code, ICD-10: C21.0-C21.8).

^**^ Cervical cancer, CIS and dysplasia include cancer, cervix (ICD-7: 1711, 1712, ICD-10: C53), CIS, cervix (ICD-7: 5710, 5711, ICD-10: D06), and dysplasia, cervix (ICD-7: 5712, ICD-10: N87).

^***^ All HPV-related disease includes cancer, head and neck, subset (ICD-7: 1410, 1411, 1418, 1450, 1442, 1458, 1480, ICD-10: C01.9, C02.4, C02.8, C09.0, C09.1, C09.8, C09.9, C10.2, C10.8, C10.9, C14.0, C14.2, C14.8), cancer, vulva and vagina (ICD-7: 1760, 1761, 1762, 1763, 1765, ICD-10: C51, C52), cancer, penis (ICD-7: 1790, ICD-10: C60), cancer, anus (ICD-7: No sufficiently specific ICD-7 code, ICD-10: C21.0-C21.8), cancer, cervix (ICD-7: 1711, 1712, ICD-10: C53), CIS, cervix (ICD-7: 5710, 5711, ICD-10: D06), and dysplasia, cervix (ICD-7: 5712, ICD-10: N87).

Abbreviations: CI, confidence interval; HR, hazard ratio, CIS, carcinoma in situ; HPV, human papillomavirus; *FLG*, filaggrin gene.

For both the logistic and Cox regression analyses, only participants with complete information on all considered variables were included. In model 1, we adjusted for gender, study population and age (not in the Cox regression analyses, since age was underlying time axis and thus accounted for). In model 2, we further adjusted for education, physical activity, smoking habits, alcohol intake, body mass index, systolic and diastolic blood pressure, serum triglycerides and HDL-cholesterol. There were no statistically significant interactions between *FLG* genotype and neither study population nor gender.

## Results

*FLG* mutation status was not associated with the baseline characteristics except for statistically significantly higher levels of HDL-cholesterol in the Allergy98 and the Inter99 study (table 1). Table 2 displays the covariates according to an all-time HPV-related diagnosis of cancer or pre-cancer. As expected due to the contribution of cervical cancers and pre-cancers, a HPV-related diagnosis is significantly associated with female gender in crude analyses. In addition and also in crude analyses, an all-time HPV-related diagnosis is significantly associated with younger age, lower BMI, daily smoking, lower alcohol consumption, lower systolic and diastolic blood pressure, lower triglycerides and higher HDL-cholesterol (table 2).

Table 3 shows the distribution of prevalent and incident cancers and pre-cancers in the 4 studies. For HPV-related cancer in particular, the number of events in some of the categories is very low (table 3). In table 4, the associations between *FLG* genotype and prevalent and all-time HPV-related cancers and pre-cancer are shown. With a total of 534 events, the association between *FLG* genotype and all HPV-related disease (all-time) was statistically significant with an odds ratio, OR = 1.4 (95% confidence interval, CI: 1.0, 1.8) for *FLG* loss-of-function mutation carriers when adjusted for study, gender, age, education, physical activity, smoking habits, alcohol intake, BMI, systolic and diastolic blood pressure, triglycerides and cholesterol (table 4, model 2). The remaining associations were statistically non-significant although a few were borderline significant (table 4).

Table 5 shows the associations between *FLG* genotype and incident HPV-related cancer and pre-cancer. The median (min, max) follow-up time for all HPV-related cancer and pre-cancer was 11.5 (0.1, 18.1) years, and the person-years-at-risk was 137,725 years. The associations between *FLG* genotype and cervical cancer, CIS and dysplasia, and all HPV-related disease were statistically significant with hazard ratio, HR = 2.0 (95% CI: 1.0, 4.0) and HR = 2.1 (95% CI: 1.2, 3.7), respectively, for *FLG* mutation carriers vs. *FLG* wild type in the fully adjusted model (table 5, model 2). In general, the associations remained essentially unchanged by multiple adjustments.

## Discussion

*FLG* mutation carrier status was significantly associated with a higher risk of incident cervical cancer and pre-cancer and all HPV-related cancers and pre-cancers in Danish adults. Furthermore, a significantly higher risk was found among *FLG* mutation carriers of prevalent or incident HPV-related cancer or pre-cancer. To our knowledge, no study has investigated this before.

Although previous histological studies on filaggrin and the cervix aimed to evaluate the use of filaggrin expression as a diagnostic criteria in cervical lesion, the conclusions are somewhat in line with our results: Cintorino et al found that filaggrin expression was more irregular in the high risk HPV type cervical lesions (HPV 16 and 18) as compared to the low risk (HPV 6, 11 and 31) [25]. Lara et al found that filaggrin expression could serve as a marker of differentiation in both normal and pathological cervical tissue and that even neoplastic lesion may have regular filaggrin expression if well differentiated [26]. Thus, the above-mentioned studies suggest that disturbed filaggrin expression is more often seen in less differentiated pre-cancers and cancers.

*FLG* mutation carrier status could affect the risk of HPV-related cancer and pre-cancer in several ways. First, since the skin is an important barrier against microorganisms, impaired skin barrier function caused by filaggrin deficiency may lead to a greater susceptibility to microorganisms [7]. Hence, Mildner et al found that mutation carriers had impaired filament aggregation and a reduced number of tight junctions in a skin model [11]. The absence of filaggrin resulted in a higher UV sensitivity, likely due to a smaller amount of epidermal urocanic acid, a breakdown product of the filaggrin molecule. As a possible consequence, *FLG* mutations are associated with up to 10% higher levels of vitamin D, possibly due to higher UV sensitivity [27], [28]. Also, a hydrophilic fluorescent dye easily penetrated through the stratum corneum down to the basal layer of the filaggrin-deficient skin cultures [11]. Of note, a recent study found that filaggrin knockdown did not affect either epidermal morphogenenesis, lipid organization in stratum corneum, lipid composition, or the lipophilic permeability of stratum corneum in a skin equivalent [29]. They concluded that *FLG* knockdown alone may not necessarily affect the skin barrier function [29]. However, atopic dermatitis affects approximately 40% of *FLG* mutation carriers and is characterized by a skin barrier defect and an increased susceptibility to cutaneous microorganism colonization and infection [7]: the skin of persons with atopic dermatitis are frequently colonized by *S. aureus*, and atopic dermatitis is rather frequently complicated by both localized and disseminated cutaneous viral infections such as herpes simplex virus, HPV, or molluscum virus [30].

Second, elevated pH of the stratum corneum due to less acidic degradation products of filaggrin may lead to increased adhesion of microorganisms [7]. Thus, Miajlovic et al found the principal breakdown products of filaggrin to slow the growth of *Staphylococcus (s.) aureus*, suggesting that *FLG* mutation carriers who have less filaggrin breakdown products may favor *S. aureus* susceptibility [31]. In addition, Gao et al found a higher risk of eczema herpeticum in *FLG* mutation carriers with atopic dermatitis compared with *FLG* wild types with atopic dermatitis [32].

Third, a low grade skin inflammation can promote the conditions for neoplastic cells to proliferate thus increasing the risk of local cancer [12]-[14]: inflammation contributes to proliferation and survival of malignant cells, angiogenesis and metastasis, and induction of genetic instability with accumulated random genetic changes in cancer cells [13].

Further research into a potential effect of *FLG* loss-of-function mutations on other microorganism related cancers is important. Other bacteria and viruses known to be carcinogenic are hepatitis B and C virus (hepatocarcinoma); Epstein-Barr virus (lymphoma and nasopharyngeal carcinoma); and helicobacter pylori (gastric cancer) [12], [33]. Also, suspected to be carcinogenic are *salmonella typhi* (carcinoma of the gallbladder), *streptococcus bovis* (colorectal cancer), and *chlamydia pneumonia* (lung cancer) [12], [33].

The strengths of our study include the prospective design and the large population-based samples; a long-term follow-up and the use of standardised registry-based diagnoses with a high degree of completeness and a minimal loss to follow-up. Using a genetic marker such as *FLG* also establishes the time sequence i.e. that the exposure happens before the outcome, eliminating the risk of reverse causation. As shown in table 1, *FLG* mutations were not related to several factors that may be related to cancer risk suggesting that the observed association between *FLG* mutations and HPV-related cancer and pre-cancer risk is not mediated by pleiotropic effects of *FLG* mutations on these factors. The validity of the cancer diagnoses in the Danish Cancer Registry is high with the proportion of morphologically verified tumors of 89% [21], a validity secured through daily quality control routines and in completing the yearly publication of the Cancer Registry and the quality achieved by e.g. manual coding of complex cases [21]. HPV is considered mandatory for cervical cancer development whereas the fraction of cancers caused by HPV differ among other HPV-related cancer types. We included only the cancers which we consider most strongly associated with HPV [24].

The limitations of the study include the relatively low number of HPV-related cancers; possible delay from onset of disease until inclusion in the register; and the risk of selection bias/survivor bias if the mortality differs between the *FLG* wildtype and mutations carriers before study participation e.g. if *FLG* mutation carriers die younger. However, we recently showed that *FLG* mutation carrier status was not associated with mortality [20]. Also, the proportion of cancer caused by HPV differs between the included cancer types [1], and this may have led to some misclassification which has likely attenuated the observed associations. The attenuation is however probably small since cervical cancers and pre-cancers are by far the largest contributor here and these are almost entirely HPV-related [1].

We found that *FLG* mutation carrier status was significantly associated with a higher incidence of cervical cancer and pre-cancer and all HPV-related cancers and pre-cancers. Our data suggest that *FLG* mutation carriers are at particular risk of HPV-related cancer and pre-cancer and therefore likely to benefit more from preventive measures such as vaccines and screening. Our results need to be interpreted with caution due to the presumably low filaggrin expression in some of the affected tissues and the relatively low number of cases. We, however, hypothesize that the excess risk of HPV-related cancers and pre-cancers among *FLG* mutation carriers is caused by an impaired epidermal skin barrier due to *FLG* deficiency that leads to increased retention and penetration of HPV in tissue with eventual cancer development.
